# Tetra­aqua­bis[2-(thio­semi­carbazono­methyl)benzene­sulfonato]manganese(II)

**DOI:** 10.1107/S160053680904495X

**Published:** 2009-10-31

**Authors:** Xi-Shi Tai, Zeng-Bing Zhao

**Affiliations:** a College of Chemistry and Chemical Engineering, Weifang University, Weifang 261061, People’s Republic of China; b Department of Chemistry, Qinghai Normal University, Xining 810008, People’s Republic of China

## Abstract

In the title compound, [Mn(C_8_H_8_N_3_O_3_S_2_)_2_(H_2_O)_4_], the Mn^II^ atom (site symmetry 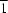) adopts a slightly distorted octa­hedral MnO_6_ geometry. The mol­ecular conformation is supported by N—H⋯N and O—H⋯O hydrogen bonds. In the crystal, mol­ecules inter­act by O—H⋯O, O—H⋯S, N—H⋯O and N—H⋯S hydrogen bonds, thereby forming (011) sheets.

## Related literature

For background to coordination networks, see: Ranford *et al.* (1998 ▶).
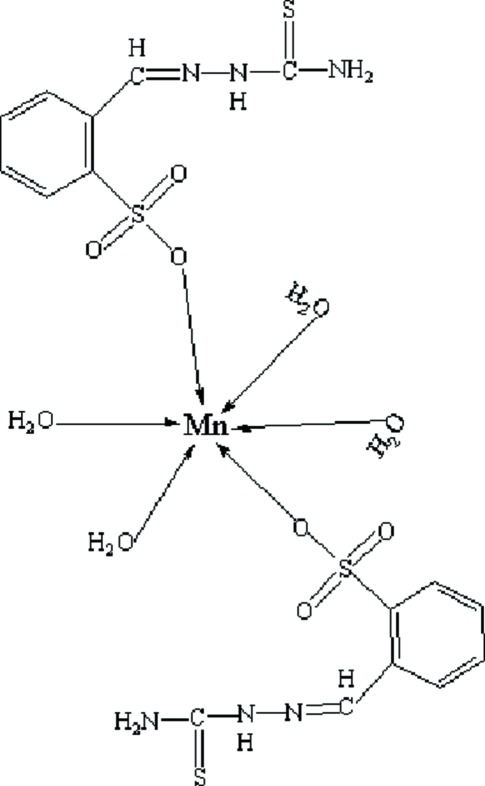


## Experimental

### 

#### Crystal data

[Mn(C_8_H_8_N_3_O_3_S_2_)_2_(H_2_O)_4_]
*M* *_r_* = 643.59
Triclinic, 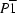

*a* = 6.8096 (6) Å
*b* = 9.5498 (8) Å
*c* = 10.7898 (9) Å
α = 64.386 (1)°
β = 88.495 (1)°
γ = 83.791 (1)°
*V* = 628.83 (9) Å^3^
*Z* = 1
Mo *K*α radiation
μ = 0.92 mm^−1^
*T* = 273 K
0.19 × 0.14 × 0.12 mm

#### Data collection

Bruker SMART CCD diffractometer
Absorption correction: multi-scan (*SADABS*; Bruker, 2000 ▶) *T*_min_ = 0.845, *T*_max_ = 0.898
3329 measured reflections
2207 independent reflections
2005 reflections with *I* > 2σ(*I*)
*R*_int_ = 0.015

#### Refinement

*R*[*F* ^2^ > 2σ(*F* ^2^)] = 0.028
*wR*(*F* ^2^) = 0.077
*S* = 1.07
2207 reflections
170 parameters
H-atom parameters constrained
Δρ_max_ = 0.36 e Å^−3^
Δρ_min_ = −0.34 e Å^−3^

### 

Data collection: *SMART* (Bruker, 2000 ▶); cell refinement: *SAINT* (Bruker, 2000 ▶); data reduction: *SAINT*; program(s) used to solve structure: *SHELXS97* (Sheldrick, 2008 ▶); program(s) used to refine structure: *SHELXL97* (Sheldrick, 2008 ▶); molecular graphics: *SHELXTL* (Sheldrick, 2008 ▶); software used to prepare material for publication: *SHELXTL*.

## Supplementary Material

Crystal structure: contains datablocks global, I. DOI: 10.1107/S160053680904495X/hb5192sup1.cif

Structure factors: contains datablocks I. DOI: 10.1107/S160053680904495X/hb5192Isup2.hkl

Additional supplementary materials:  crystallographic information; 3D view; checkCIF report

## Figures and Tables

**Table 1:** Selected bond lengths (Å)

Mn1—O4	2.1369 (17)
Mn1—O5	2.1495 (16)
Mn1—O1	2.2166 (14)

**Table 2:** Hydrogen-bond geometry (Å, °)

*D*—H⋯*A*	*D*—H	H⋯*A*	*D*⋯*A*	*D*—H⋯*A*
N1—H1*A*⋯N3	0.86	2.29	2.638 (3)	104
O4—H10⋯O3	0.85	2.02	2.761 (3)	146
N1—H1*B*⋯O3^i^	0.86	2.33	2.986 (3)	134
N2—H2⋯S2^ii^	0.86	2.57	3.4231 (19)	170
O4—H9⋯S2^iii^	0.85	2.36	3.182 (2)	162
O5—H11⋯S2^iv^	0.85	2.46	3.2603 (19)	156
O5—H12⋯O2^v^	0.85	1.87	2.712 (3)	172

Symmetry codes: (i) 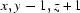; (ii) 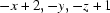; (iii) 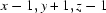; (iv) 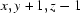; (v) 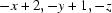.

**Fig. 1.**
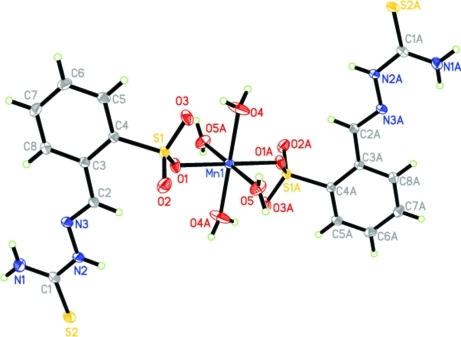
The molecular structure of (I) showing 30% displacement ellipsoids. Atoms with the suffix A are generated by the symmetry operation (1-x, 1-y, -z).

## supplementary crystallographic information

### Experimental

A solution of 1.0 mmol 2-formyl-benzenesulfonate-thiosemicarbazide was added to a solution of 0.5 mmol MnCl_2_.4H_2_O in 5 ml ethanol at room temperature. The mixture was refluxed for 4 h with stirring, then the resulting precipitate was filtered, washed, and dried *in vacuo* over P_4_O_10_ for 48 h. Pink blocks of (I) were obtained by slowly evaporating from methanol at room temperature.

### Refinement

The H atoms were positioned geometrically (C—H = 0.93, N—H = 0.86, O—H = 0.85Å) and refined as riding with *U*_iso_(H)= 1.2 *U*_eq_(C, N) or 1.5*U*_eq_(O).

### Crystal data

**Table:**

[Mn(C_8_H_8_N_3_O_3_S_2_)_2_(H_2_O)_4_]	*Z* = 1
*M**_r_* = 643.59	*F*(000) = 331
Triclinic, *P*1	*D*_x_ = 1.700 Mg m^−^^3^
Hall symbol: -P 1	Mo *K*α radiation, λ = 0.71073 Å
*a* = 6.8096 (6) Å	Cell parameters from 2123 reflections
*b* = 9.5498 (8) Å	θ = 2.4–28.2°
*c* = 10.7898 (9) Å	µ = 0.92 mm^−^^1^
α = 64.386 (1)°	*T* = 273 K
β = 88.495 (1)°	Block, pink
γ = 83.791 (1)°	0.19 × 0.14 × 0.12 mm
*V* = 628.83 (9) Å^3^	

### Data collection

**Table:**

Bruker SMART CCD diffractometer	2207 independent reflections
Radiation source: fine-focus sealed tube	2005 reflections with *I* > 2σ(*I*)
graphite	*R*_int_ = 0.015
ω scans	θ_max_ = 25.1°, θ_min_ = 2.1°
Absorption correction: multi-scan (*SADABS*; Bruker, 2000)	*h* = −8→8
*T*_min_ = 0.845, *T*_max_ = 0.898	*k* = −11→11
3329 measured reflections	*l* = −9→12

### Refinement

**Table:**

Refinement on *F*^2^	Secondary atom site location: difference Fourier map
Least-squares matrix: full	Hydrogen site location: inferred from neighbouring sites
*R*[*F*^2^ > 2σ(*F*^2^)] = 0.028	H-atom parameters constrained
*wR*(*F*^2^) = 0.077	*w* = 1/[σ^2^(*F*_o_^2^) + (0.0384*P*)^2^ + 0.3206*P*] where *P* = (*F*_o_^2^ + 2*F*_c_^2^)/3
*S* = 1.07	(Δ/σ)_max_ = 0.001
2207 reflections	Δρ_max_ = 0.36 e Å^−^^3^
170 parameters	Δρ_min_ = −0.34 e Å^−^^3^
0 restraints	Extinction correction: *SHELXL97* (Sheldrick, 2008), Fc^*^=kFc[1+0.001xFc^2^λ^3^/sin(2θ)]^-1/4^
Primary atom site location: structure-invariant direct methods	Extinction coefficient: 0.049 (3)

### Special details

**Table:**

Geometry. All e.s.d.'s (except the e.s.d. in the dihedral angle between two l.s. planes) are estimated using the full covariance matrix. The cell e.s.d.'s are taken into account individually in the estimation of e.s.d.'s in distances, angles and torsion angles; correlations between e.s.d.'s in cell parameters are only used when they are defined by crystal symmetry. An approximate (isotropic) treatment of cell e.s.d.'s is used for estimating e.s.d.'s involving l.s. planes.
Refinement. Refinement of *F*^2^ against ALL reflections. The weighted *R*-factor *wR* and goodness of fit *S* are based on *F*^2^, conventional *R*-factors *R* are based on *F*, with *F* set to zero for negative *F*^2^. The threshold expression of *F*^2^ > σ(*F*^2^) is used only for calculating *R*-factors(gt) *etc*. and is not relevant to the choice of reflections for refinement. *R*-factors based on *F*^2^ are statistically about twice as large as those based on *F*, and *R*- factors based on ALL data will be even larger.

### Fractional atomic coordinates and isotropic or equivalent isotropic displacement parameters (Å^2^)

**Table:**

	*x*	*y*	*z*	*U*_iso_*/*U*_eq_	
Mn1	0.5000	0.5000	0.0000	0.02389 (16)	
S1	0.73935 (8)	0.67535 (6)	0.15413 (5)	0.02518 (16)	
S2	1.00275 (9)	−0.13908 (6)	0.71686 (6)	0.03340 (18)	
O1	0.6334 (2)	0.54631 (17)	0.16197 (15)	0.0333 (4)	
O2	0.9517 (2)	0.6407 (2)	0.15616 (17)	0.0417 (4)	
O3	0.6673 (3)	0.82183 (18)	0.04065 (15)	0.0386 (4)	
O4	0.3965 (3)	0.7431 (2)	−0.0981 (2)	0.0652 (6)	
H9	0.2884	0.7905	−0.1404	0.098*	
H10	0.4415	0.7985	−0.0637	0.098*	
O5	0.7698 (2)	0.5559 (2)	−0.11007 (18)	0.0433 (4)	
H12	0.8605	0.4911	−0.1165	0.065*	
H11	0.8157	0.6433	−0.1363	0.065*	
N1	0.8828 (3)	0.0620 (2)	0.81969 (19)	0.0400 (5)	
H1A	0.8452	0.1545	0.8118	0.048*	
H1B	0.8919	−0.0160	0.8996	0.048*	
N2	0.9104 (3)	0.16402 (19)	0.58726 (18)	0.0271 (4)	
H2	0.9474	0.1543	0.5143	0.033*	
N3	0.8342 (2)	0.30737 (19)	0.57900 (17)	0.0257 (4)	
C1	0.9263 (3)	0.0400 (2)	0.7097 (2)	0.0269 (5)	
C2	0.8091 (3)	0.4167 (2)	0.4577 (2)	0.0242 (4)	
H2A	0.8437	0.3975	0.3821	0.029*	
C3	0.7252 (3)	0.5734 (2)	0.4382 (2)	0.0216 (4)	
C4	0.6849 (3)	0.6967 (2)	0.3074 (2)	0.0212 (4)	
C5	0.6053 (3)	0.8429 (2)	0.2938 (2)	0.0284 (5)	
H5A	0.5780	0.9236	0.2066	0.034*	
C6	0.5664 (3)	0.8694 (3)	0.4078 (2)	0.0345 (5)	
H6	0.5140	0.9679	0.3979	0.041*	
C7	0.6056 (3)	0.7491 (3)	0.5373 (2)	0.0329 (5)	
H7	0.5791	0.7667	0.6147	0.040*	
C8	0.6835 (3)	0.6034 (3)	0.5523 (2)	0.0283 (5)	
H8	0.7090	0.5235	0.6401	0.034*	

### Atomic displacement parameters (Å^2^)

**Table:**

	*U*^11^	*U*^22^	*U*^33^	*U*^12^	*U*^13^	*U*^23^
Mn1	0.0270 (3)	0.0197 (2)	0.0237 (3)	−0.00156 (17)	−0.00312 (17)	−0.00820 (19)
S1	0.0331 (3)	0.0220 (3)	0.0199 (3)	−0.0055 (2)	0.0001 (2)	−0.0080 (2)
S2	0.0437 (3)	0.0198 (3)	0.0306 (3)	0.0006 (2)	−0.0044 (2)	−0.0057 (2)
O1	0.0521 (10)	0.0266 (8)	0.0230 (8)	−0.0120 (7)	−0.0042 (7)	−0.0104 (6)
O2	0.0352 (9)	0.0540 (11)	0.0448 (10)	−0.0042 (8)	0.0067 (7)	−0.0301 (9)
O3	0.0603 (11)	0.0259 (8)	0.0221 (8)	−0.0100 (7)	−0.0032 (7)	−0.0020 (7)
O4	0.0675 (13)	0.0263 (9)	0.0932 (16)	0.0101 (9)	−0.0507 (12)	−0.0182 (10)
O5	0.0405 (10)	0.0403 (10)	0.0559 (11)	−0.0125 (8)	0.0177 (8)	−0.0262 (9)
N1	0.0639 (14)	0.0245 (10)	0.0246 (10)	−0.0001 (9)	0.0020 (9)	−0.0051 (8)
N2	0.0313 (9)	0.0206 (9)	0.0239 (9)	0.0028 (7)	−0.0010 (7)	−0.0056 (7)
N3	0.0269 (9)	0.0203 (9)	0.0267 (10)	−0.0011 (7)	−0.0023 (7)	−0.0074 (8)
C1	0.0250 (11)	0.0235 (11)	0.0261 (11)	−0.0033 (8)	−0.0033 (8)	−0.0047 (9)
C2	0.0263 (10)	0.0228 (10)	0.0225 (10)	−0.0017 (8)	−0.0020 (8)	−0.0091 (9)
C3	0.0206 (10)	0.0211 (10)	0.0238 (10)	−0.0031 (8)	−0.0027 (8)	−0.0100 (8)
C4	0.0217 (10)	0.0212 (10)	0.0216 (10)	−0.0043 (8)	−0.0016 (8)	−0.0095 (8)
C5	0.0320 (11)	0.0194 (10)	0.0308 (12)	−0.0016 (9)	−0.0024 (9)	−0.0080 (9)
C6	0.0375 (13)	0.0260 (12)	0.0450 (14)	0.0009 (10)	−0.0004 (10)	−0.0210 (11)
C7	0.0362 (12)	0.0367 (13)	0.0361 (12)	−0.0053 (10)	0.0022 (10)	−0.0250 (11)
C8	0.0306 (11)	0.0297 (11)	0.0240 (11)	−0.0039 (9)	−0.0013 (9)	−0.0108 (9)

### Geometric parameters (Å, °)

**Table:**

Mn1—O4^i^	2.1369 (17)	N1—H1B	0.8600
Mn1—O4	2.1369 (17)	N2—C1	1.337 (3)
Mn1—O5^i^	2.1495 (16)	N2—N3	1.376 (2)
Mn1—O5	2.1495 (16)	N2—H2	0.8600
Mn1—O1	2.2166 (14)	N3—C2	1.274 (3)
Mn1—O1^i^	2.2166 (14)	C2—C3	1.469 (3)
S1—O2	1.4468 (17)	C2—H2A	0.9300
S1—O3	1.4489 (16)	C3—C8	1.396 (3)
S1—O1	1.4647 (15)	C3—C4	1.403 (3)
S1—C4	1.7767 (19)	C4—C5	1.388 (3)
S2—C1	1.702 (2)	C5—C6	1.374 (3)
O4—H9	0.8500	C5—H5A	0.9300
O4—H10	0.8499	C6—C7	1.381 (3)
O5—H12	0.8499	C6—H6	0.9300
O5—H11	0.8499	C7—C8	1.376 (3)
N1—C1	1.313 (3)	C7—H7	0.9300
N1—H1A	0.8600	C8—H8	0.9300
			
O4^i^—Mn1—O4	180.0	H1A—N1—H1B	120.0
O4^i^—Mn1—O5^i^	88.04 (8)	C1—N2—N3	119.42 (18)
O4—Mn1—O5^i^	91.96 (8)	C1—N2—H2	120.3
O4^i^—Mn1—O5	91.96 (8)	N3—N2—H2	120.3
O4—Mn1—O5	88.04 (8)	C2—N3—N2	115.48 (18)
O5^i^—Mn1—O5	180.0	N1—C1—N2	118.31 (19)
O4^i^—Mn1—O1	92.72 (7)	N1—C1—S2	122.79 (16)
O4—Mn1—O1	87.28 (7)	N2—C1—S2	118.89 (16)
O5^i^—Mn1—O1	92.43 (6)	N3—C2—C3	119.54 (19)
O5—Mn1—O1	87.57 (6)	N3—C2—H2A	120.2
O4^i^—Mn1—O1^i^	87.28 (7)	C3—C2—H2A	120.2
O4—Mn1—O1^i^	92.72 (7)	C8—C3—C4	117.81 (18)
O5^i^—Mn1—O1^i^	87.57 (6)	C8—C3—C2	119.86 (18)
O5—Mn1—O1^i^	92.43 (6)	C4—C3—C2	122.33 (18)
O1—Mn1—O1^i^	180.0	C5—C4—C3	120.37 (18)
O2—S1—O3	113.09 (10)	C5—C4—S1	117.47 (15)
O2—S1—O1	112.81 (10)	C3—C4—S1	122.14 (15)
O3—S1—O1	111.89 (9)	C6—C5—C4	120.6 (2)
O2—S1—C4	105.67 (9)	C6—C5—H5A	119.7
O3—S1—C4	106.74 (9)	C4—C5—H5A	119.7
O1—S1—C4	105.98 (9)	C5—C6—C7	119.6 (2)
S1—O1—Mn1	131.56 (9)	C5—C6—H6	120.2
Mn1—O4—H9	131.3	C7—C6—H6	120.2
Mn1—O4—H10	115.1	C8—C7—C6	120.3 (2)
H9—O4—H10	108.1	C8—C7—H7	119.8
Mn1—O5—H12	126.2	C6—C7—H7	119.8
Mn1—O5—H11	124.0	C7—C8—C3	121.2 (2)
H12—O5—H11	108.1	C7—C8—H8	119.4
C1—N1—H1A	120.0	C3—C8—H8	119.4
C1—N1—H1B	120.0		
			
O2—S1—O1—Mn1	−96.18 (14)	C8—C3—C4—S1	177.75 (15)
O3—S1—O1—Mn1	32.68 (16)	C2—C3—C4—S1	−2.0 (3)
C4—S1—O1—Mn1	148.66 (12)	O2—S1—C4—C5	115.31 (17)
O4^i^—Mn1—O1—S1	138.91 (14)	O3—S1—C4—C5	−5.33 (19)
O4—Mn1—O1—S1	−41.09 (14)	O1—S1—C4—C5	−124.75 (17)
O5^i^—Mn1—O1—S1	−132.94 (13)	O2—S1—C4—C3	−62.83 (18)
O5—Mn1—O1—S1	47.06 (13)	O3—S1—C4—C3	176.53 (15)
O1^i^—Mn1—O1—S1	−25 (100)	O1—S1—C4—C3	57.11 (18)
C1—N2—N3—C2	−174.55 (18)	C3—C4—C5—C6	0.6 (3)
N3—N2—C1—N1	−5.3 (3)	S1—C4—C5—C6	−177.58 (17)
N3—N2—C1—S2	174.98 (14)	C4—C5—C6—C7	−0.5 (3)
N2—N3—C2—C3	178.94 (17)	C5—C6—C7—C8	0.2 (3)
N3—C2—C3—C8	3.4 (3)	C6—C7—C8—C3	0.1 (3)
N3—C2—C3—C4	−176.81 (18)	C4—C3—C8—C7	0.0 (3)
C8—C3—C4—C5	−0.3 (3)	C2—C3—C8—C7	179.78 (19)
C2—C3—C4—C5	179.89 (19)		

Symmetry codes: (i) −*x*+1, −*y*+1, −*z*.

### Hydrogen-bond geometry (Å, °)

**Table:**

*D*—H···*A*	*D*—H	H···*A*	*D*···*A*	*D*—H···*A*
N1—H1A···N3	0.86	2.29	2.638 (3)	104
O4—H10···O3	0.85	2.02	2.761 (3)	146
N1—H1B···O3^ii^	0.86	2.33	2.986 (3)	134
N2—H2···S2^iii^	0.86	2.57	3.4231 (19)	170
O4—H9···S2^iv^	0.85	2.36	3.182 (2)	162
O5—H11···S2^v^	0.85	2.46	3.2603 (19)	156
O5—H12···O2^vi^	0.85	1.87	2.712 (3)	172

Symmetry codes: (ii) *x*, *y*−1, *z*+1; (iii) −*x*+2, −*y*, −*z*+1; (iv) *x*−1, *y*+1, *z*−1; (v) *x*, *y*+1, *z*−1; (vi) −*x*+2, −*y*+1, −*z*.
